# Spatiotemporal control of necroptotic cell death and plasma membrane recruitment using engineered MLKL domains

**DOI:** 10.1038/s41420-022-01258-0

**Published:** 2022-11-29

**Authors:** Amir Taslimi, Kaiah M. Fields, Kristin D. Dahl, Qi Liu, Chandra L. Tucker

**Affiliations:** Department of Pharmacology, Box 8303, University of Colorado School of Medicine, Aurora, CO 80045 USA

**Keywords:** Necroptosis, Synthetic biology

## Abstract

Necroptosis is a form of programmed necrotic cell death in which a signaling cascade induces oligomerization of mixed lineage kinase domain-like (MLKL) protein, leading to plasma membrane rupture. Necroptotic cell death is recognized as important for protection against viral infection and has roles in a variety of diseases, including cancer and diabetes. Despite its relevance to health and disease states, many questions remain about the precise mechanism of necroptotic cell death, cellular factors that can protect cells from necroptosis, and the role of necroptosis in disease models. In this study, we engineered a light-activated version of MLKL that rapidly oligomerizes and is recruited to the plasma membrane in cells exposed to light, inducing rapid cell death. We demonstrate this tool can be controlled spatially and temporally, used in a chemical genetic screen to identify chemicals and pathways that protect cells from MLKL-induced cell death, and used to study signaling responses of non-dying bystander cells. In additional studies, we re-engineered MLKL to block its cell-killing capacity but retain light-mediated membrane recruitment, developing a new single-component optogenetic tool that allows modulation of protein function at the plasma membrane.

## Introduction

Necroptosis is a form of regulated cell death mediated by receptor-interacting protein kinases (RIPK1/RIPK3), which induce plasma membrane (PM) rupture through activation of mixed lineage kinase domain-like (MLKL) [1, 2]. Necroptotic cell death is implicated in a variety of health and disease states, such as viral infection, cancer, and diabetes, among other diseases [3–6]. During necroptosis, MLKL is stimulated by RIPK3 to oligomerize and localize to the PM, cumulating in membrane rupture. Oligomerization allows PM recruitment of MLKL, leading to cell permeabilization through the formation of pores, although the precise molecular mechanism of this process remains under debate [7]. Issues still under debate include the precise oligomerization state of MLKL [7], a potential role in auxiliary proteins for membrane recruitment [8], and the role of accessory proteins or calcium influx in membrane permeabilization [7, 9–12].

To explore the important roles and mechanism of necroptosis, chemical tools targeting MLKL have been developed, including necrosulfonamide [1], which blocks necroptosis, and chemical dimerizers that directly activate MLKL by inducing oligomerization [13]. While chemical dimerizer approaches are effective, chemicals can have off-target effects, act slowly, and lack spatial control. To allow a more precise study of the mechanism and consequences of necroptotic death, we developed an optogenetic approach to induce MLKL-mediated necroptosis using cryptochrome 2 (CRY2), an *Arabidopsis* photoreceptor that oligomerizes upon blue light stimulation [14–16]. Using this approach, we triggered the recruitment of MLKL to the PM and cell permeabilization upon blue light exposure. In the course of this work, we became aware of two other studies also using CRY2 to activate MLKL, using different MLKL domains and approaches [17, 18]. We compared the three strategies in side-by-side experiments, finding differences in background activity and rate of cell death. In further work, we explored the mechanism of MLKL-induced death and cellular factors that can protect cells after induction of MLKL activation. Using a chemical genetics approach with light-triggered MLKL, we identified compounds affecting calcium homeostasis, among other targets, that protect from cell death. Finally, we applied our knowledge to engineer a modified light-triggered MLKL that translocates to the PM but does not kill the cell, providing an efficient way to regulate protein activity at the PM with light.

## Results

### An MLKL_1–140_-CRY2 fusion induces necroptosis with light but shows background activity

**Fig. 1:**
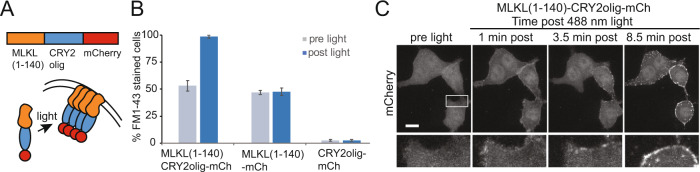
Initial validation of light-induced cell death using MLKL(1–140)-CRY2olig fusion. **A** Schematic showing overview of construct and strategy. **B** Quantification of FM1-43 staining in HEK293T cells expressing MLKL(1–140)-CRY2olig-mCh, MLKL(1–140), or CRY2-mCh (negative control). MLKL-expressing cells show FM1–43 staining (indicating cell permeabilization) in 100% of expressing cells with light, but also substantial membrane leakage in the dark. The graph shows the average and range of the two experiments. **C** Representative HEK293T cells expressing MLKL(1–140)-CRY2olig-mCh before and after blue light exposure (images show mCherry fluorescence). Light exposure leads to the recruitment and clustering of MLKL at the plasma membrane, leading to pronounced cell rounding and death. Scale bar, 10 µm.

Human MLKL is comprised of a 4HB executionary domain, linked to a regulatory pseudokinase domain by a two-helix brace. In its inactive state, the 4HB domain is packed tightly against the pseudokinase domain, holding cell death activity in check [19]. Activation involves RIPK3-mediated phosphorylation, thought to lead to a conformation that exposes the 4-HB domain and results in oligomerization and PM recruitment, however, the mechanistic details of this process remain incompletely understood [1, 7, 8, 13, 20]. To generate a light-responsive MLKL, we modified a strategy previously used with chemical dimerizers [13], attaching residues 1–140 of the 4HB domain to CRY2olig, which undergoes blue light-inducible oligomerization [15], to generate MLKL_1–140_-CRY2olig-mCh (Fig. 1A). Upon expression in HEK293T cells, we observed high numbers of dead cells even without inducing oligomerization, while cells that did survive typically showed low MLKL expression. Of these viable cells, ~50% showed loss of PM integrity even without light exposure, as monitored by staining with the steryl fluorescent dye FM1-43, which exhibits increased fluorescence upon binding lipid in the internal cellular environment [21] (Fig. 1B). After illumination, 100% of cells expressing MLKL-CRY2olig-mCh showed loss of PM integrity, compared to 2% of cells expressing a CRY2olig-mCh control. For cells that remained viable in the dark, illumination with a brief (200 ms) pulse of 488 nm light resulted in clustering and PM recruitment of MLKL-CRY2olig-mCh, followed by cell rounding and death (Fig. 1C, Supplementary Video 1). These results indicated that we could achieve light-inducible cell death, but that this approach required improvements to prevent the significant background observed.

### Improved light control of cell death using FLAG-MLKL-CRY2olig

To achieve tighter control of MLKL, we explored factors contributing to cell death in the uninduced state. Replacing CRY2olig with other CRY2 versions that show differences in the degree of oligomerization [15, 22] had no effect on background cell death, as monitored by propidium iodide (PI), a red fluorescent probe that labels permeabilized nuclei, while cells expressing MLKL_1–140_ without CRY2 (MLKL_1–140_-EGFP) showed significantly elevated death rates compared with EGFP (Supplementary Fig. S1). These results suggested that overexpression of MLKL_1-140_, rather than CRY2 self-association, was causing background cell death.

**Fig. 2:**
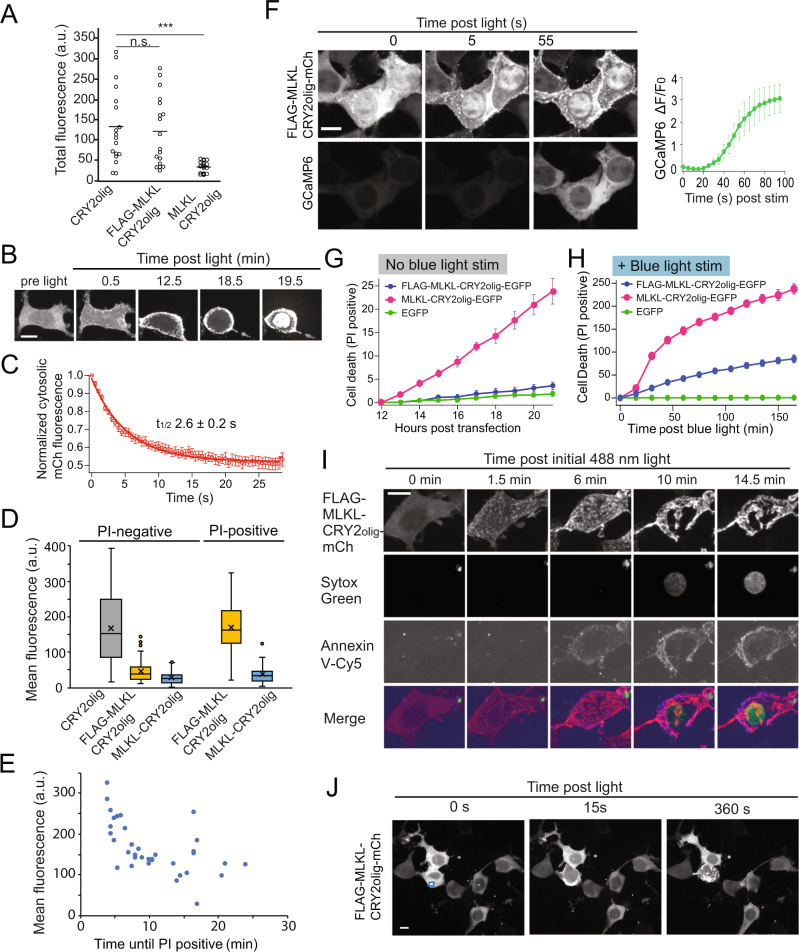
FLAG-MLKL allows improved light control of cell death. **A** Comparison of expression of MLKL-CRY2olig-mCh (‘MLKL-CRY2olig’) and FLAG-MLKL-CRY2olig-mCh in HEK293T cells. Cells were transfected using Lipofectamine 2000, and mCherry fluorescence was quantified 24 h post-transfection. Control cells expressing CRY2olig-mCherry were also quantified for comparison. a.u., arbitrary units; n.s., *p*-value = 0.70; ***, *p*-value < 0.001, two-tailed unpaired *t*-test. **B** Representative images of cells expressing FLAG-MLKL before and after light exposure. HEK293T cells transfected with FLAG-MLKL-CRY2olig-mCh were incubated with 1 µg/ml PI and exposed to blue light (100 ms pulse, 488 nm, every 30 s). FLAG-MLKL translocates to the plasma membrane and results in cell rounding. PI staining can be observed as a sudden appearance of nuclear fluorescence, seen here at 19.5 min. **C** Quantification of loss of cytosolic FLAG-MLKL upon light-induced recruitment to the PM. HEK293T cells expressing FLAG-MLKL-CRY2olig-mCh were exposed to blue light 24 h after transfection. A large fraction of FLAG-MLKL translocates from the cytosol to the plasma membrane with a half-life of 2.6 s. Data represents mean ± s.e.m. of six cells from three independent experiments. **D** Correlation of expression level and cell fate. Graph shows mean total fluorescence of HEK293T cells expressing indicated constructs exposed to a single pulse of blue light and monitored for 30 min for PI-positive staining. (*a.u., arbitrary units*). *n* = 60–175 cells quantified per construct. Data are representative of *n* = 3 independent experiments. **E** Graph showing the relationship between expression level and time until cell death (PI staining), for cells treated as in (**D**) (35 cells quantified). Data are representative of *n* = 3 independent experiments. **F** Representative images (left) and quantification (right) of HEK293T cells expressing FLAG-MLKL-CRY2olig-mCh and GCaMP6. Cells show a large increase in GCaMP6 signal within 60 s after light exposure. Graph shows mean ± s.e.m., *n* = 6 cells. **G** HEK293T cells expressing indicated constructs and incubated with 1 µg/ml PI were quantified for cell death (PI-positive staining) in the absence of blue light stimulation using an IncuCyte imager. Graph shows mean ± s.e.m. of triplicate samples. Data are representative of *n* = 2 independent experiments. **H** Cells expressing indicated constructs incubated with PI and exposed to light (488 nm pulse every 15 min) were quantified for cell death (PI staining) using an IncuCyte imager. Data represent mean and s.e.m. of quadruplicate samples, and are representative of *n* = 3 independent experiments. **I** Representative confocal images of HEK293T cells expressing FLAG-MLKL exposed to blue light. Cells also contained 400 nM Sytox green and Cy5-Annexin V. **J** Focal application of blue light to a single cell expressing FLAG-MLKL-CRY2olig-mCh. Blue light was applied only to the area indicated by the blue box using an Andor Mosaic digital mirror device, while the entire region was imaged using the mCherry channel. Scale bar (all images), 10 μm.

To reduce background, we modified the N-terminus of MLKL, as previous studies had indicated that the fusion of polypeptides at its N-terminus can hinder its necroptotic function [20, 23]. In initial tests, we used mCherry-tagged versions and appended an N-terminal FLAG tag (FLAG-MLKL_1-140_-CRY2olig-mCh or ‘FLAG-MLKL’). In contrast to MLKL-CRY2olig-mCh, which we were unable to observe expressed at high levels, FLAG-MLKL accumulated to high levels in cells incubated in the dark, comparable to a CRY2olig-mCh control (Fig. 2A). After light exposure, ~50% of cells expressing FLAG-MLKL showed rapid clustering of MLKL and translocation to the PM, followed by cell rounding and permeabilization (resulting in nuclear PI staining) (Fig. 2B, Supplementary Video 2). PM recruitment occurred rapidly, within 10 s after light exposure (*t*_1/2_ = 2.6 s) (Fig. 2C). Cell fate was tightly correlated with expression level: nearly all (97%) of the cells above a threshold concentration showed PI-positive staining within 30 min, whereas below that concentration, nearly all (98%) cells remained PI-negative (Fig. 2D). For cells that died, PI staining occurred between 3 and 24 min after light onset (Fig. 2E), however, expression alone did not accurately predict the timing of PI appearance, indicating other factors at play. Using a GCaMP6 Ca^2+^ sensor, we observed a large increase in intracellular Ca^2+^ within tens of seconds after light addition (Fig. 2F, Supplementary Video 3), similar to previous results with chemical-dimerized MLKL systems [11].

To quantify cell death over a larger time frame and population, we used EGFP-tagged proteins and monitored the appearance of PI-stained cells, which can be visualized without exposing cells to blue light that stimulates CRY2. In HEK293T cells expressing FLAG-MLKL and exposed only to 561 nm light for imaging mCherry (no blue light exposure), we observed minimal death, indicating low leakiness of this construct in the unstimulated condition (Fig. 2G). In contrast, cells expressing FLAG-MLKL showed a robust increase in cell death (as quantified by PI-staining) upon exposure to blue light, delivered at 20 h post-transfection (Fig. 2H). Cells expressing FLAG-MLKL showed concurrent Cy5-Annexin V-staining (indicative of phosphatidylserine surface exposure) and Sytox green nuclear staining, consistent with death by necroptosis [24] (Fig. 2I, Supplementary Video 4). Focal light application to select cells expressing FLAG-MLKL led to PM recruitment of MLKL followed by cell rounding only in illuminated cells, without affecting non-light-treated cells (Fig. 2J, Supplementary Video 5).

### Investigating cellular responses to damage from light-stimulated MLKL

Prior studies have demonstrated the survival of cells that have activated necroptosis [11, 25], indicating that stimulation of this pathway does not always result in cell death. One protein complex important for cell survival during necroptosis is ESCRT-III, which is recruited to sites of cell permeabilization and mediates the repair of damaged membranes, delaying cell death [25, 26]. Cells that experience PM damage due to MLKL activation but do not immediately proceed to cell death have been found to activate a PKC signaling pathway leading to cytokine and chemokine production [27], the release of which can activate immune surveillance mechanisms.

**Fig. 3:**
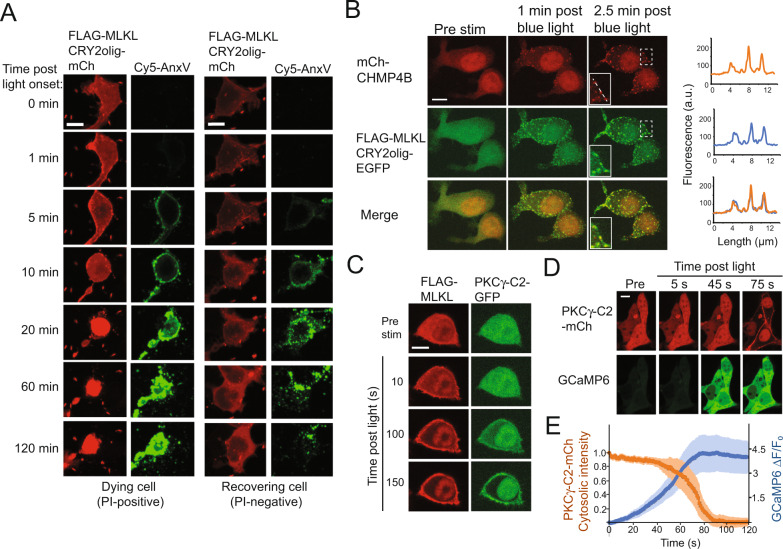
Cellular response to light-stimulated MLKL damage. **A** Representative images of HEK293T cells expressing FLAG-MLKL-CRY2olig-mCh and exposed to blue light showing staining with Cy5-Annexin V and 1 µg/ml PI. Cells were exposed to a mild light stimulus (single, 150 ms pulse of 488 nm light) and tracked for 120 min. The majority of cells that showed Annexin V after light treatment ultimately underwent cell death, represented by PI staining in the mCherry channel (the two left panels show an example of these cells). Approximately 9% of cells that showed Annexin V staining after light treatment did not show PI staining and recovered (right panel). Scale bars, 10 µm. **B** Representative HEK293T cells showing colocalization of FLAG-MLKL-CRY2olig-EGFP and mCherry-CHMP4B on the PM after light stimulation. The graph at right shows the quantification of fluorescence intensity along the dotted white line. Scale bar, 10 µm. **C** Representative images of cells expressing FLAG-MLKL-CRY2olig-mCh and PKCγ-C2-GFP. PKCγ-C2-GFP is recruited to the PM within 100 s after light-triggered MLKL stimulation. Scale bar, 10 µm. **D**, **E** Dual visualization of Ca^2+^ and PKC-C2γ responses following FLAG-MLKL stimulation. Representative images (**D**) and quantification (**E**) of cells expressing FLAG-MLKL-CRY2olig-miRFP and the sensors PKCγ-C2-mCh and GCaMP6 before and after blue light stimulation (100 ms pulse of 488 nm light). The orange graph shows the change in normalized cytosolic fluorescence intensity of mCherry signal over time in cells expressing PKCγ-C2-mCh. The blue graph shows GCaMP6 signal (∆*F*/*F*_0_) change over time. An increase in intracellular Ca^2+^ is observed ~40 s after light application, followed by recruitment of C2γ-mCh to the PM. Quantification is from *n* = 5 cells from two independent experiments. Scale bar, 10 µm.

To examine cell responses to MLKL activation, we first looked at whether cells expressing FLAG-MLKL could be induced (by sub-saturating light exposure) to activate the necroptosis pathway but not die (Fig. 3A). While most cells that became Annexin V-positive could not recover, with 89% ultimately becoming PI-positive during the course of imaging (Fig. 3A, left panel), ~9% of cells that stained positive for Annexin V demonstrated the ability to recover (Fig. 3A, right panel), initially showing Annexin V-positive staining that diminished over time, with no appearance of PI.

These studies indicated that light-treated cells could undergo the initial steps of necroptosis, including Annexin V exposure, without dying. Using a mCh-CHMP4B sensor to visualize ESCRT-III [25], we observed co-clustering of mCh-CHMP4B and FLAG-MLKL-CRY2olig-EGFP upon light stimulation (Fig. 3B). FLAG-MLKL activation was also sufficient to activate PKCγ, as monitored by PM translocation of a PKCγ-C2-GFP sensor [27, 28] (Fig. 3C, Supplementary Video 6). Recruitment of C2γ occurred ~45 s after light onset, and 20 s after initial changes in Ca^2+^ could be observed (Fig. 3D, E). These results show that activation of FLAG-MLKL recapitulates similar responses to damage as observed using other approaches to activate MLKL [27].

### Discriminating cell-autonomous (autocrine) vs non-cell-autonomous (paracrine) signaling events

**Fig. 4:**
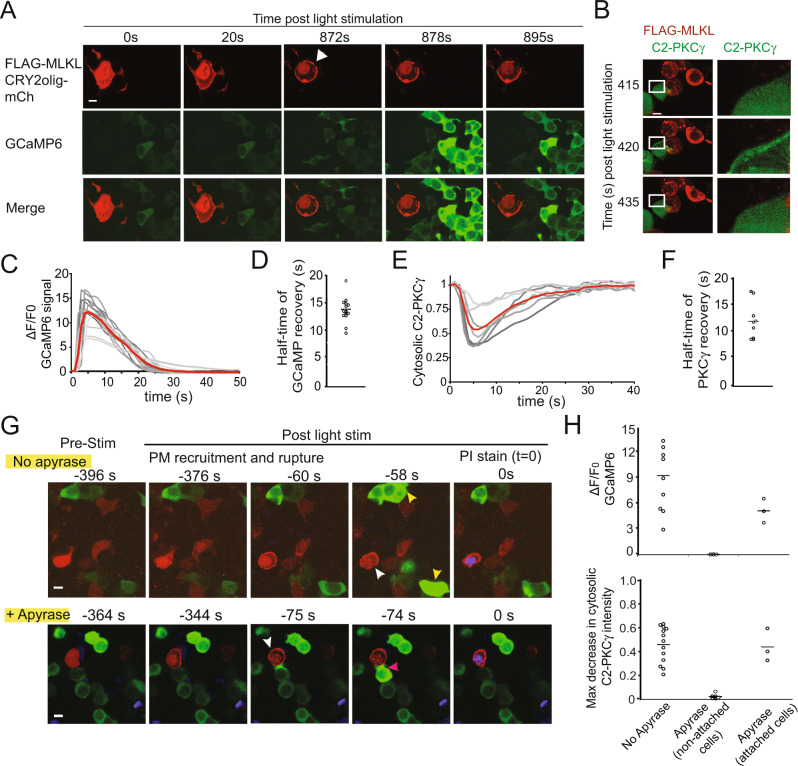
FLAG-MLKL-induced cell death induces signaling to bystander cells. **A** Representative images of HEK293T cells were separately transfected with FLAG-MLKL-CRY2olig-mCh or GCaMP6, then mixed at a ratio of 1:5, respectively. Minutes after light stimulation, FLAG-MLKL-expressing cells show membrane rupture (white arrow). Within 6 s after rupture, the surrounding GCaMP6-expressing bystander cells show an increase in Ca^2+^. **B** Representative images of HEK293T cells separately transfected with FLAG-MLKL-CRY2olig-mCh or PKCγ-C2-GFP, then mixed at a ratio of 1:5. Minutes after light onset, FLAG-MLKL-induced cell death (rounded cell on right side of left panel) triggers transient activation and membrane recruitment of PKC in bystander cells. The zoom of the area in a white rectangle is shown on the right. **C** Quantification of change in GCaMP signal (∆*F*/*F*_0_) in bystander cells in proximity to dying Flag-MLKL-expressing cell as in (**A**). Time 0 marks the beginning of rise in GCaMP signal, as defined by an increase in baseline > 2 standard deviations from the mean. Thirteen cells were quantified from *n* = 3 independent experiments. **D** Quantification of the half-time of recovery from peak GCaMP signal from responding cells in (**C**). **E** Quantification of change in cytosolic intensity of PKCγ-C2-GFP in bystander cells in proximity to dying FLAG-MLKL-expressing cells as in (**B**). Shown are eight cells from *n* = 3 independent experiments. **F** Quantification of the half-time of recovery from peak cytosolic depletion of PKCγ-C2-GFP, for cells shown in (**E**). **G**, **H** Apyrase addition blocks Ca^2+^ and PKCγ responses in unattached bystander cells. The experiment was performed as in (A), except cells were incubated with or without 10 U/ml apyrases and used a far-red MLKL (FLAG-MLKL-CRY2olig-miRFP670_nano_) to simultaneously assess MLKL membrane recruitment, nuclear PI staining, and either GCaMP6 or C2γ-GFP signal. Upon cell rupture (white arrowheads), nearby GCaMP-expressing observer cells (yellow arrowheads) show an increase in Ca^2+^ in the absence, but not presence, of apyrase. Cells that are attached to the dying cell (pink arrows) were not affected by apyrase treatment. Representative images are shown in (**G**), with quantification of the GCaMP (max ∆*F*/*F*_0_) and PKCγ (max cytosolic decrease) responses in bystander cells within 150 µm in (**H**). Data were obtained from 3 independent experiments. Scale bar, 10 μm (all images).

Studies have shown that cells dying through necroptosis release DAMPs (damage-associated molecular patterns) that can signal to neighbor cells [29, 30]. As DAMPs can induce spikes in intracellular Ca^2+^ in neighboring cells [31], we wanted to confirm that the Ca^2+^ changes we observed in Fig. 2F were due to cell-autonomous processes, rather than DAMPs. To observe DAMP signaling, we co-cultured cells expressing FLAG-MLKL (dying cells) with cells that were separately transfected with GCaMP6 (bystander cells). Upon light activation, MLKL-expressing cells showed rapid PM translocation of MLKL, followed minutes later by the appearance of a membrane breach (Fig. 4A). Several seconds later (872 s in Fig. 4A), a Ca^2+^ spike could be observed in bystander cells (Fig. 4A, Supplementary Video 7). Using a far-red labeled FLAG-MLKL, we observe the timing of the neighboring cell Ca^2+^ spike just prior to the appearance of PI nuclear stain within the dying cell (Supplementary Fig. S2). In parallel experiments, we observed membrane translocation of a PKC-C2γ-GFP sensor in bystander cells (Fig. 4B). Both the spike in Ca^2+^ and the C2γ-GFP recruitment were transient (*t*_1/2_ of GCaMP6 recovery = 13.9 ± 2.2 s; *t*_1/2_ of C2γ cytosolic recovery = 12.9 ± 2.2 s) (Fig. 4C–F; Supplementary Video 8).

To identify the DAMP(s) responsible for the Ca^2+^ and PKC responses, we carried out the same imaging experiment performed in Fig. 4A, but in the presence or absence of the ATP-hydrolyzing enzyme apyrase [31]. Apyrase did not block the Ca^2+^ increase in physically attached bystander cells, possibly due to the fast diffusion of ATP at close proximity, but did block the rise in Ca^2+^ and translocation of PKC in physically separated bystander cells (Fig. 4G, H). The Ca^2+^ spikes due to DAMP signaling were distinguishable, due to their transient nature, from the sustained rise in Ca^2+^ we observed in dying (MLKL-expressing) cells, which we confirmed was not blocked by apyrase (Supplementary Fig. S3).

### Screen for compounds that protect cells from light-mediated cell death

**Fig. 5:**
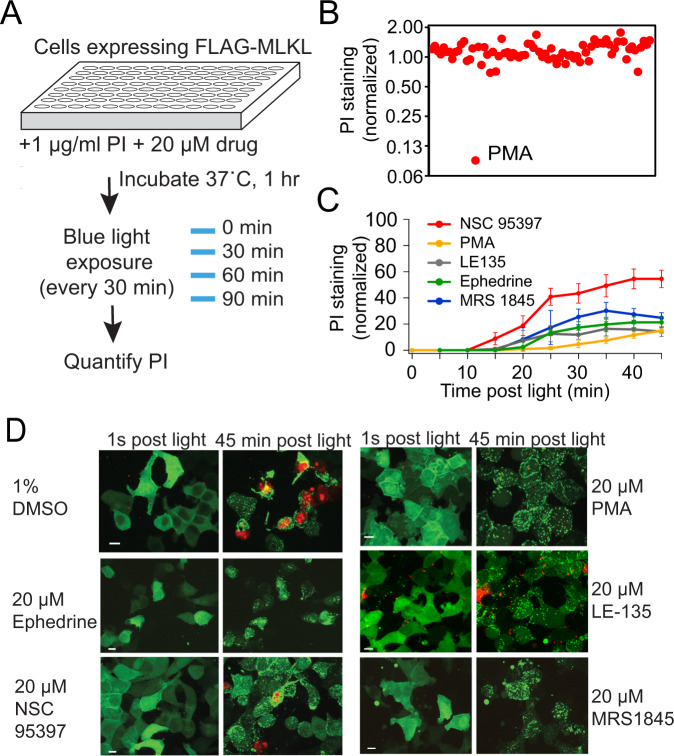
Screen for compounds that protect against light-triggered cell death. **A** Schematic of the screen. HEK293T cells expressing FLAG-MLKL-CRY2olig-EGFP were incubated with 20 µM of each compound for 1 h, along with 1 µg/ml PI, then exposed to blue light (every 30 min for 90 min). The ratio of PI staining to EGFP expression was quantified after 90 min of light treatment. **B** Normalized PI staining results from one 96-well plate. The positive hit PMA, which results in a reduced level of PI staining, is shown. **C** Retests of primary hit compounds from the screen. HEK293T cells expressing FLAG-MLKL were incubated with 20 µM of indicated compounds, exposed to light, then quantified for PI stain. Data are expressed as a percent of DMSO control at the final time point (45 min). Graph shows mean ± s.d., *n* = 3 replicates, from two experiments for NSC95397, PMA, LE135, and MRS1845, and one experiment for ephedrine hemisulfate. **D** Representative images of MLKL (green) and PI staining (red) in cells 1 s and 45 min post light exposure, in cells treated with indicated chemicals as in (**C**). Scale bars, 10 µm.

We envisioned that light-triggered FLAG-MLKL could provide a unique system for further probing necroptosis. The precise mechanism of cell killing, and whether accessory proteins are required, is still under debate. It is also not fully understood how cells circumvent a death signal. Compounds that prolong cell viability in the face of a death signal, allowing activation of signaling leading to the release of cytokines [27], could be useful for enhancing innate immune responses in disease models. To explore these concepts, we initiated a chemical genetic screen using a Sigma LOPAC (Library of Pharmacologically Active Compounds) library to identify compounds that protect cells from MLKL-induced death, using light as an activator (Fig. 5A, B). As a positive control, we included 2-aminoethoxydiphenyl borate (2-APB), a modulator of calcium signaling that was previously found to protect from necroptosis in HT29 cells activated through the native signaling pathway [9]. Of 1053 compounds initially screened, 12 showed a decrease in cell death (Table 1). Hits included several that directly modulate Ca^2+^ entry, consistent with prior results indicating that Ca^2+^ plays a physiologically important role [9, 11]. In addition to 2-APB, we identified MRS-1845, a blocker of store-operated Ca^2+^ channels, and FPL 64176, an activator of L-type Ca^2+^ channels. Five other hits were found to modulate Ca^2+^ homeostasis, though through more indirect methods or having a range of other targets, including phorbol 12-myristate 13-actate (PMA), ouabain and dihydroouabain, GW7647, and niclosamide [32–34]. Two compounds that affect signaling cascades, NSC-95397 and ephedrine sulfate, were also identified. A detailed summary of the screen is provided in Supplementary Table 1.

**Table 1:** Summary of hit compounds protecting cells from light-triggered MLKL activity.

		Normalized PI stain (% of DMSO control)	
Drug name	Target/mechanism of action	Trial 1	Trial 2
LOPAC screen			
2-APB (100 µm, positive control)	Ca2+ channel blocker	1.8	4.2
Ebselen	Antioxidant inhibits lipid peroxidation	14.8	8.9
FPL 64176	Activator of L-type Ca^2+^ channels	9.6	17.5
Phorbol 12-myristate 13-acetate (PMA)	PKC activator	14.0	21.4
Ouabain	Inhibitor of Na+/K+ ATPase, activates signaling pathways	19.3	9.1
Dihydroouabain	Inhibitor of Na+/K+ ATPase	36.3	58.3
MRS 1845	The blocker of store-operated Ca^2+^ channels	15.6	17.8
NSC 95397	Cdc25 dual specificity phosphatase inhibitor	19.0	2.9
(−)-ephedrine hemisulfate	Alpha and beta-adrenergic agonist	19.2	11.2
3,4-dichloroisocoumarin	Serine protease inhibitor	39.6	7.3
N-succinyl-L-proline		27.5	26.3
GW7647	Activator of PPARalpha affects lipid peroxidation and acid-sensing ion channels	19.7	9.2
Niclosamide	Uncouples oxidative phosphorylation, activates TMEM16A (Ca2+-gated anion channel) [32], and inhibits SERCA pump and Ca^2+^ influx channels.	48.8	30.5
MedChemExpress Screen			
LE135	RARbeta antagonist, activates TRP channels	25.1	12.1
PYR6	Orai1 channel blocker also blocks TRPC3 with lower affinity	16.8	53.9
PYR10	TRPC3 blocker	12.3	33.1
TPC2-A1-P	Two pores Ca^2+^ channel agonist	10.1	17.2

To better understand the impact of calcium regulation on MLKL-induced cell death, we screened 162 additional compounds with roles in modulating ion channels and calcium signaling (MedChemExpress), and obtained four additional hits (Table 1). Among these were PYR6 and PYR10, which modulate Orai and TRPC channels [35], and LE135, a TRP channel activator [36]. In a previous study, knockdown of the TRPM7 channel had been found to be protective against necroptosis [9]. A fourth hit was TPC2-A1-P, an agonist of two-pore Ca^2+^ channels [37]. Using confocal imaging to verify the initial screen results, we observed that cells incubated with PMA, NSC-95397, LE135, MRS1845, or ephedrine showed significantly reduced PI staining at 45 min compared with a DMSO control (Fig. 5C, D, Supplementary Fig. S4). We examined whether chemical addition could block MLKL-induced Ca^2+^ influx, but saw no difference in light-triggered GCaMP6 fluorescence compared with DMSO controls for cells treated with PMA, 2-APB, or MRS1845 (Supplementary Fig. S5). Interestingly, cells treated with these three compounds all showed enhanced membrane bleb formation (seen at 180 s in Supplementary Fig. S5).

### Engineering a non-killing version of MLKL-CRY2olig for light-dependent PM recruitment

Based on our studies with the N-terminal FLAG tag, we wondered if the placement of a larger protein at the N-terminus could prevent cell killing while maintaining membrane recruitment. Such a module could be used to recruit cargo to the PM with light. While there exist other tools for light-dependent PM recruitment, most require two components: a hook protein permanently anchored at the PM, and a protein that inducibly binds the hook. One exception is a single-component system based on the photoreceptor BcLOV4, which undergoes a light-dependent protein-lipid electrostatic interaction allowing PM recruitment of a variety of cargo [38]. We explored whether we could generate a similar single-component membrane recruitment tool using MLKL.

**Fig. 6:**
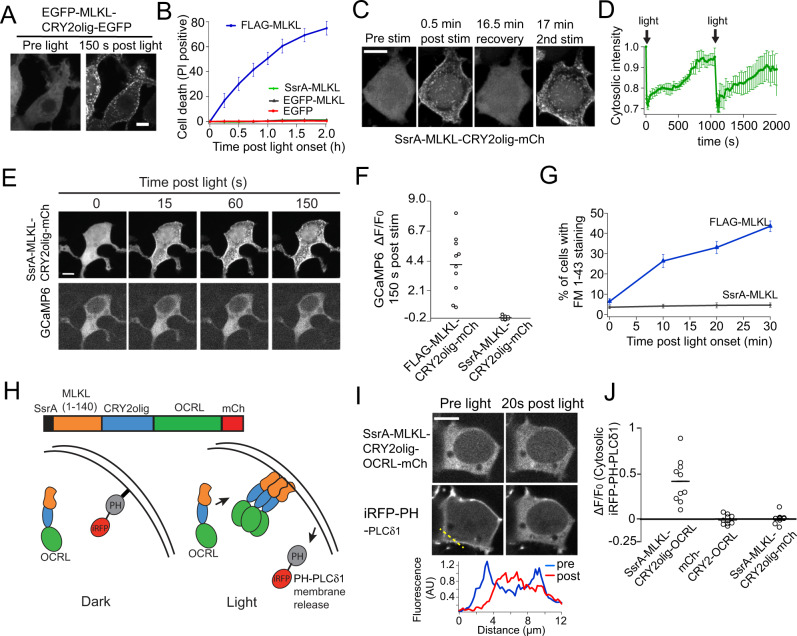
Engineering a non-killing version of MLKL-CRY2olig that allows light-dependent recruitment to the plasma membrane. **A** Representative images of HEK293T cells expressing EGFP-MLKL-CRY2olig-EGFP before and 150 s after exposure to 488 nm light. MLKL is rapidly recruited to the PM. **B** Quantification of light-triggered death (PI staining) in cells expressing indicated MLKL-CRY2olig constructs. Fusion of EGFP or SsrA at the N-terminus of MLKL-CRY2olig-EGFP blocks triggered cell death. The graph depicts mean ± s.e.m., *n* = 3 independent experiments. **C** Representative images of cells expressing SsrA-MLKL-CRY2olig-mCh, before and after 488 nm light delivered at 0 and 16.5 min, with a 16.5 min period of recovery in between light stimulations. **D** Quantification of change in cytosolic levels of SsrA-MLKL-CRY2olig-mCh in the experiment as in (**C**). (*n* = 6 cells quantified from two independent experiments, mean ± s.e.m.) **E**, **F** Representative images (**E**) and quantification (**F**) of cells expressing SsrA-MLKL-CRY2olig-mCh and GCaMP6 and exposed to light. No increase in GCaMP6 intensity is observed despite the recruitment of SsrA-MLKL to the plasma membrane. Cells expressing FLAG-MLKL, for comparison in (**F**), show a large increase in GCaMP6 signal. The graph shows *n* = 10 cells quantified from 2 independent experiments. **G** Percent of cells expressing FLAG-MLKL or SsrA-MLKL showing FM1-43 staining after light stimulation. Graph shows mean ± s.e.m. from three independent experiments. **H** Schematic showing strategy for recruiting SsrA-MLKL-CRY2olig-OCRL to PM with light. Recruitment results in the translocation of a PH-PLCδ1 sensor from the PM to the cytosol. **I** Representative images of cells expressing SsrA-MLKL-CRY2olig-OCRL-mCh and iRFP-PH-PLCδ1 before and after light induction. The graph below shows the relative fluorescence intensity of iRFP-PH-PLCδ1 at the dotted white line before and after blue light. **J** Quantification of fold change in cytosolic iRFP-PH-PLCδ1 intensity [(Intensity_post_ − Intensity_pre_)/Intensity_pre_] 20 s after light induction in cells expressing indicated OCRL fusions and control constructs. All scale bars, 10 µm.

To develop such a tool, we swapped the FLAG with EGFP to generate EGFP-MLKL-CRY2olig-EGFP, which was cytosolic in the dark, translocated to the PM with blue light (Fig. 6A), but showed minimal to no cell death in dark or after illumination (Fig. 6B). To toggle killing ability on or off with binding of a second protein, we also tested a peptide (MAANDENR; SsrA) that binds a larger protein, SspB [39], however, SsrA-MLKL on its own effectively blocked killing (Fig. 6B). SsrA-MLKL-CRY2olig-mCh could be repeatedly recruited to the PM (Fig. 6C, D; Supplementary Video 9) and showed no change in intracellular Ca^2+^ (Fig. 6E, F) or membrane integrity (Fig. 6G) upon light stimulation.

To demonstrate the utility of SsrA-MLKL as a standalone membrane recruitment tool, we tested the use of the system to recruit the inositol 5-phophatase domain of OCRL to the PM, resulting in dephosphorylation of PI(4,5)P_2_, an approach previously developed using a two-component CRY2-CIB optogenetic system [40]. We fused SsrA-MLKL to the inositol 5-phophatase domain of OCRL (SsrA-MLKL-CRY2olig-OCRL) and tracked OCRL activity using a sensor, iRFP-PH PLCδ1, that translocates from the PM to the cytosol upon loss of PI(4,5)P_2_ [40] (Fig. 6H). With light, SsrA-MLKL-CRY2olig-OCRL translocated to the PM and displaced PH-PLCδ1 from the PM (Fig. 6I, J), while no displacement of PH-PLCδ1 was observed with modules lacking MLKL or OCRL (Fig. 6J).

### Comparison of different optogenetic MLKL systems

**Fig. 7:**
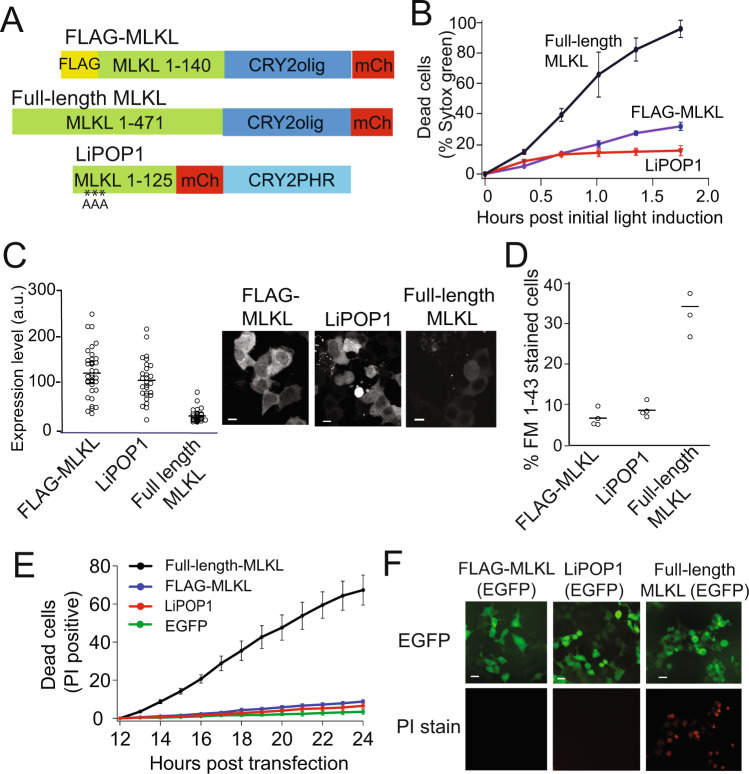
Comparison of light-activated MLKL versions. **A** Schematic showing constructs compared in the study. **B** HEK293T cells expressing indicated mCherry-tagged MLKL constructs were quantified for cell death (Sytox green positive staining) in response to light (488 nm light every 20 min for 100 min). Graph shows mean ± s.e.m, *n* = 3 independent experiments. **C** Quantification of mCherry fluorescence within cells expressing indicated MLKL constructs described in (**A**), with representative images at right. Scale bars, 10 µm. **D** Quantification of FM1–43 staining in cells expressing mCherry-tagged versions of MLKL and incubated in the dark. Data represents mean ± s.e.m. for three independent experiments. **E** Quantification of background cell death in the dark (absence of blue light). EGFP-tagged versions of indicated MLKL versions (or EGFP as a control) were quantified for cell death (positive PI-staining) over time in the dark. Data are expressed as a percent of cells with PI-positive stain, out of total MLKL expressing cells, and represents mean ± s.e.m., *n* = 3. **F** Representative images of cells expressing EGFP-tagged FLAG-MLKL, LiPOP1, or full-length MLKL at 23 h post-transfection and kept in the dark. Full-length MLKL samples accumulated substantial levels of PI-positive staining in the dark. Scale bars, 20 µm.

While preparing this paper, we became aware of several other studies describing alternate versions of light-activated MLKL. In one approach, a circularly-permuted LOV2 photosensory domain was used to regulate the activity of a 1–178 aa N-terminal fragment of MLKL (MLKL-cpLOV2) [41]. While the tool was minimally characterized, induction of cell death was slow, occurring several hours after light induction. In an independent study from the same lab, researchers used a similar approach as we used (light-induced clustering of CRY2) to generate ‘LiPOP1’, comprising residues 1–125 of MLKL fused to CRY2PHR (N-terminal CRY2) [18]. As in our study, they found that the use of unmodified, truncated MLKL resulted in significant unstimulated cell death, which they reduced by mutating three residues (Y15A/E16A/R17A). A third study also used CRY2 clustering, fusing CRY2olig to full-length MLKL [17]. As the tools using CRY2 to induce MLKL were evaluated using different platforms and expression systems, we undertook a systematic comparison of their killing behavior and kinetics, along with our FLAG-MLKL tool. We expressed FLAG-MLKL, ‘LiPOP1’ (MLKL_1–125_-CRY2PHR-mCh [18], or ‘full-length-MLKL’ (MLKL_1–471_-CRY2olig-mCh) [17] in HEK293T cells, and used Sytox green to quantify cell death (Fig. 7A). After 100 min blue light exposure, full-length-MLKL showed robust killing, with 98.8% of cells Sytox green-positive; FLAG-MLKL and LiPOP1, in turn, showed lower levels of Sytox staining (FLAG-MLKL, 29.4%; LiPOP1, 15.3%), consistent with their engineered attenuated killing abilities (Fig. 7B).

While high light-induced killing behavior is desirable in a triggered cell death tool, a useful optogenetic tool will also show minimal cell death in the uninduced (dark) state. For example, our initial construct, MLKL(1-140)-CRY2olig-mCh, showed robust light-induced killing but induced such high levels of cell death in the dark as to preclude its use. We suspected that the full-length-MLKL construct may show substantial death in the uninduced (dark) state since, prior to light activation, the relative expression level of full-length-MLKL was very low compared to cells expressing LiPOP1 or FLAG-MLKL (Fig. 7C), reminiscent of our initial studies with MLKL(1–140)-CRY2olig-mCh (Fig. 1) and suggesting that high-expressing cells were dying. Consistent with these results, we found that even in the dark (without blue light exposure), ~33% of cells expressing full-length-MLKL showed compromised membrane integrity, as assayed using FM1–43 permeabilization assay, compared with 6% for FLAG-MLKL and 9% for LiPOP1 (Fig. 7D). To investigate this further, we quantified background death in cells expressing each MLKL version over time in the dark. To monitor dead cells without exposure to blue light, we used EGFP-tagged MLKL versions and PI staining, which could be followed without blue light exposure, as in Fig. 2G. Cells expressing full-length-MLKL showed substantially higher background levels of cell death, compared to cells expressing FLAG-MLKL, LiPOP1, or EGFP (Fig. 7E, F). Taken together, our results suggest that while full-length-MLKL-CRY2olig-mCh shows robust light-induced killing, the system suffers from high background cell death and altered membrane integrity in the uninduced state, which will be problematic for users.

## Discussion

In this work, we developed a photoactivatable MLKL, FLAG-MLKL, that can be used to induce necroptotic cell death. We find that FLAG-MLKL recapitulates similar downstream signaling events as chemical-activated MLKL versions [11, 25, 27], inducing an increase in intracellular Ca^2+^, membrane translocation of PKCγ, and recruitment of ESCRT proteins to sites of the damaged membrane. To explore factors protecting cells from MLKL-triggered death, we used FLAG-MLKL to trigger necroptosis in a chemical genetic screen of over 1000 compounds, identifying many hits affecting cellular Ca^2+^ homeostasis. Our results support numerous previous studies implicating both extracellular and intracellular Ca^2+^ in necroptosis, such as studies showing that a reduction in extracellular Ca^2+^ could delay the onset of necroptotic cell death [9, 11], or that HT29 cells treated with the calcium channel blockers 2-APB and LaCl_3_ or knocked down for the TRPM7 Ca^2+^ channel were protected from necroptosis [9]. A role for intracellular Ca^2+^ was also indicated in studies showing the cell-permeable Ca^2+^ chelator BAPTA-AM can reduce levels of necroptosis in MEF and NIH3T3 cells [9, 10]. Adding to this complexity are our results showing the protective effects of a variety of compounds targeting different types of Ca^2+^ channels and pores, including channel blockers as well as compounds that induce an increase in intracellular Ca^2+^. For compounds that increase intracellular Ca^2+^, Ca^2+^ exposure prior to the necroptotic insult could sensitize cells, allowing the induction of protective signaling pathways or enzymes. For example, a recent study found that a Ca^2+^-activated lipid scramblase, TMEM16F, was critical for membrane repair after exposure to pore-forming agents [42]. An increase in intracellular Ca^2+^ could also increase membrane fluidity and blebbing [43], which has been found to protect cells exposed to pore-forming agents [44, 45]. Indeed, our studies showed increased membrane blebbing upon necroptosis activation with several identified hits (Supplementary Fig. S5).

Our work also explored the use of FLAG-MLKL to examine DAMP signaling from dying cells. Cells undergoing FLAG-MLKL-induced cell death induce a transient Ca^2+^ flux and transient membrane translocation of PKCγ in bystander cells, which were primarily through the release of ATP as they were blocked by apyrase. The consequences of transient PKC stimulation are unclear. As the cellular environment can contain many dying cells, multiple rounds of DAMP signaling could additively impact PKC signaling. PKC signaling was recently shown to be involved in inducing downstream chemokine expression in cells synthetically triggered for necroptosis that did not immediately die, where robust chemokine expression in MLKL-triggered cells was observed [27]. In bystander cells co-cultured with the MLKL-triggered cells, a much smaller but reproducible amount of chemokine expression was detected [27]. In future experiments, studies with or without apyrase could be undertaken to further assess the relevance of DAMP signaling on chemokine expression.

Taking another direction, we also explored the use of MLKL-CRY2olig as a single-component optogenetic recruitment tool. Fusion of EGFP or SsrA to MLKL abolished cell killing but permitted PM translocation, supporting previous studies indicating these processes are separable and that oligomerization drives PM recruitment [8, 9]. Using SsrA-MLKL, we demonstrate robust, reversible PM recruitment, which we applied to regulate inositol phosphatase activity. While there exist other PM recruitment optogenetic tools, such as CRY2-CIBN [22] and iLIDs [46], the majority of these require two components, including a protein permanently anchored at the PM that could affect function. Another effective single-component recruitment tool is BcLOV4 [38], which works well for many applications but has been shown to be sensitive to temperature [47]. For applications in which temperature sensitivity is a concern, SsrA-MLKL can thus provide a suitable replacement.

While completing this work, two other studies were published using CRY2-based optogenetic methods to activate MLKL [17, 18]. As the other studies used different approaches, we carried out a side-by-side comparison of the methods. In our hands, the LiPOP1 system behaves similarly to FLAG-MLKL, with similar low background. One minor difference is that cells expressing LiPOP1 did not always show clear PI-positive nuclear staining, a result that may explain the lower rates of PI-staining in light (Fig. 7B) and may make quantification more difficult. We find that full-length-MLKL shows similar properties as the MLKL(1–140)-CRY2olig-mCh version we originally tested (Fig. 1), with high levels of cell death in light, but also high background death and alterations in PM integrity even in the dark. Although we designed our version of this construct based on the published work [17], we note there may be subtle differences not described in the paper, such as linker differences, that may result in different behaviors in their published version.

In summary, our work demonstrates the functionality of a new optogenetic tool, FLAG-MLKL, that has a number of advantages over chemical approaches, such as the ability to steer light to stimulate MLKL in specific cells, as well as advantages over recent optogenetic MLKL tools. In addition to use with drug libraries, as we demonstrate, FLAG-MLKL is a versatile tool that can be used with CRISPR libraries or other genetic screens. Due to its ability to trigger the final stages of necroptosis rapidly and robustly with the spatiotemporal resolution, we envision this tool will be useful in future experiments examining the mechanism and consequences of necroptotic cell death.

## Materials and methods

### Plasmids and reagents

Full sequences of plasmids generated for this article are provided in Supplementary Table 2. MLKL1–140 was amplified from MGC Human MLKL sequence-verified cDNA, GE Healthcare. Full-length MLKL (1–471) was amplified from hMLKL-Venus (Addgene #106078). All PCR reactions for cloning used Phusion polymerase (New England Biolabs). FM1–43FX was from Molecular Probes (Invitrogen). PI was from Sigma. Sytox Green was from ThermoFisher. Apyrase (9000-95-7) was from Sigma. The compound libraries used for screening were Sigma (LOPAC^®^1280 library) and MedChemExpress (custom ion channel library).

### Cell culture

HEK293T cells were maintained in Dulbecco’s modified Eagle medium (DMEM) supplemented with 10% fetal bovine serum (FBS) and 1× Penicillin–Streptomycin (Corning) at 37 °C with 5% CO_2_. Cells were transfected using calcium phosphate or Lipofectamine 2000 (Invitrogen), according to the manufacturer’s protocol. For all transfection experiments, plates were wrapped in aluminum foil immediately or up to 4 h after transfection and kept in the dark until use. Quantification of cell death and imaging experiments were performed 18-24 h post-transfection. Light-treated cells were illuminated using a custom programmable LED light source (465 nm, 1.1 mW/cm^2^) [48], or using the 488 nm channel of the imaging system (Dragonfly imaging system or IncuCyte).

### Live cell imaging

For confocal imaging experiments, cells were plated in 2 mL imaging dishes and then transfected with plasmid DNA using Lipofectamine 2000 (ThermoFisher). Cells were imaged at 33 °C in a light-protected live cell imaging chamber, using an Andor Dragonfly 301 spinning disc confocal imaging system with an Olympus IX73 base and four-line ILE laser merge module and controller. Images were acquired using a 60x UPlanSApo 1.35 NA oil objective or a 40× UPLFLN40XO oil objective and collected on a 1024 × 1024 pixel Andor iXon EM-CCD camera. Data acquisition and analysis were performed with Fusion 2.0 (Andor) and ImageJ software. Focal stimulation experiments used an Andor Mosaic digital multimirror device and Andor IQ software.

### Cell permeabilization experiments

For FM1–43 staining, cells were plated in 24-well dishes and transfected using Lipofectamine 2000. The next day, the cells were washed and incubated in FM1–43FX (5 µg/ml) in Hank’s Balanced Salt Solution (HBSS). Cells were kept in the dark or exposed to light using a LED light source. For PI staining, cells were incubated in HBSS containing 1 µg/ml PI and imaged without dye washout. For Sytox green and annexin staining, cells were washed 2× in phosphate-free HBSS, then incubated with HBSS containing 400 nM Sytox green (Thermofisher, S7020) and/or Cy5 Annexin (BD Biosciences, 559933) (used at 1:40 dilution). Cells were incubated for 20 min in the dark, then dyes were removed, and cells were washed 1× with HBSS, then placed in an imaging buffer.

### Cell death quantification in Incucyte imager

To quantify cell death, HEK293T cells were transfected with indicated GFP-labeled plasmids in 96-well culture plates. Four hours after transfection (with Lipofectamine 2000), PI was added to each well (1 µg/ml final concentration), then plates were transferred to the Incucyte Zoom imaging system for incubation and imaging overnight at 37 °C and 5% CO_2_. Cells were imaged using a 20× objective, acquiring an image once per hour for 16 h to assess cell death prior to blue light stimulation. For cells subjected to blue light treatment, at 20 h, cells were imaged with RFP, GFP, and phase every 15 min for 160 min. To quantify cell death, the appearance of PI nuclear staining in sequential images was manually counted.

### Imaging quantification

Imaging quantification was performed using ImageJ 1.45 s. Confocal image Z-stacks were compiled into a maximal projection. Cell total fluorescence (indicative of MLKL construct expression levels) in Figs. 2A & 7E was determined by quantifying the background-subtracted mean fluorescence for each cell and multiplying by the area. The mean fluorescence was quantified by subtracting the mean background fluorescence from the mean fluorescence for each cell. The cytosolic fluorescence or cytosolic intensity (Figs. 2C, 3E, 4E, and 6D) was quantified by determining the background-subtracted mean fluorescence of a region of the cytosol in each image of a time series. Calculation of GCaMP6 signal (∆*F*/*F*_0_) at different time points was calculated by subtracting the total GCAMP fluorescence within the cell at the time point minus the initial fluorescence at time 0 (*F*_0_), then dividing by initial fluorescence.

#### Drug screening

HEK 293T cells (70–90% confluency) in 10 cm dishes were transiently transfected with 5 µg FLAG-MLKL (1-140)-CRY2olig-GFP (calcium phosphate method). Cells were incubated for 4 h, washed 3x with PBS, then trypsinized and re-suspended in 15 ml of DMEM + 10% FBS with 1 µg/ml PI. 150 µl of cells were added to each well of a 96-well plate and incubated overnight. After 24 h, cells were incubated with either 20 µM of each compound or 1% DMSO (control). As a positive control for cell death prevention, we used 100 µM 2-APB. Cells were imaged every 30 min using phase contrast, red (800 ms exposure), and green (400 ms exposure) channels in the IncuCyte ZOOM (Essen BioScience) platform. IncuCyte Zoom software processing definition was set to recognize GFP expressing cells (minimum size 50 uM^2^, minimum eccentricity 0.15, minimum intensity 20) and cells staining with PI (minimum size 20 µm^2^, minimum intensity 4.5). The normalized PI staining metric was generated by calculating the PI stain at the final timepoint and subtracting the PI stain at the initial time point (t = 0), then dividing by the GFP signal (representing the amount of expressed FLAG-MLKL(1-140)-CRY2olig-GFP) at the initial time point. This PI/GFP ratio for each well was then normalized to the average PI/GFP ratio for the DMSO-treated cells on the plate to account for differences in plate transfection efficiency. Compounds that were counted as hits showed PI/GFP ratios that differed from DMSO-treated controls by at least 2.5 standard deviations from the mean in two independent experiments. A full listing of results for each well is provided in Supplementary Table 1. Results listed as ‘NA’ were initially identified as hits but eliminated as false positives after manual visual inspection of images, with the majority of false positives due to issues with the Incucyte automatic focus or compounds that were fluorescent or colored that affected the PI/GFP calculations.

### Co-culture of MLKL-expressing cells and bystander cells

HEK 293T cells were separately transfected (calcium phosphate method) with FLAG-MLKL-CRY2olig-mCh and either GCaMP6 or the C2γ-GFP sensor. After 4 h, cells were washed 3× with PBS, trypsinized, then plated on imaging dishes at a ratio of 1:3 (Flag-MLKL to sensor). After incubation overnight, cells were imaged. Cells treated with apyrase were incubated with 10 U/mL of apyrase, and added 5 min prior to imaging.

## Supplementary information

Supplementary Video 1

Supplementary Video 2

Supplementary Video 3

Supplementary Video 4

Supplementary Video 5

Supplementary Video 6

Supplementary Video 7

Supplementary Video 8

Supplementary Video 9

Supporting Information

Supplementary Table 1

Supplementary Table 2

## Data Availability

A complete dataset of the chemical screen results is provided in Supplementary Table 1. Additional datasets used in the analysis for the current study are available from the corresponding author on reasonable request.
