# Supplementary material for: Spatiotemporal control of necroptotic cell death and plasma membrane recruitment using engineered MLKL domains
Source: Cell Death Discov. 2022 Nov 29;8:469. doi: 10.1038/s41420-022-01258-0 (PMC9709077; doi:10.1038/s41420-022-01258-0)
Supplement: Supplementary file 10 — Supporting Information [file 41420_2022_1258_MOESM10_ESM.pdf]

Supporting Information

Spatiotemporal control of necroptotic cell death and plasma membrane recruitment using engineered MLKL domains.

Amir Taslimi[1], Kaiah M. Fields[1], Kristin D. Dahl[1], Qi Liu[1], and Chandra L. Tucker[1]*

[1]Department of Pharmacology, University of Colorado School of Medicine, Aurora, CO 80045

*Correspondence: chandra.tucker@cuanschutz.edu

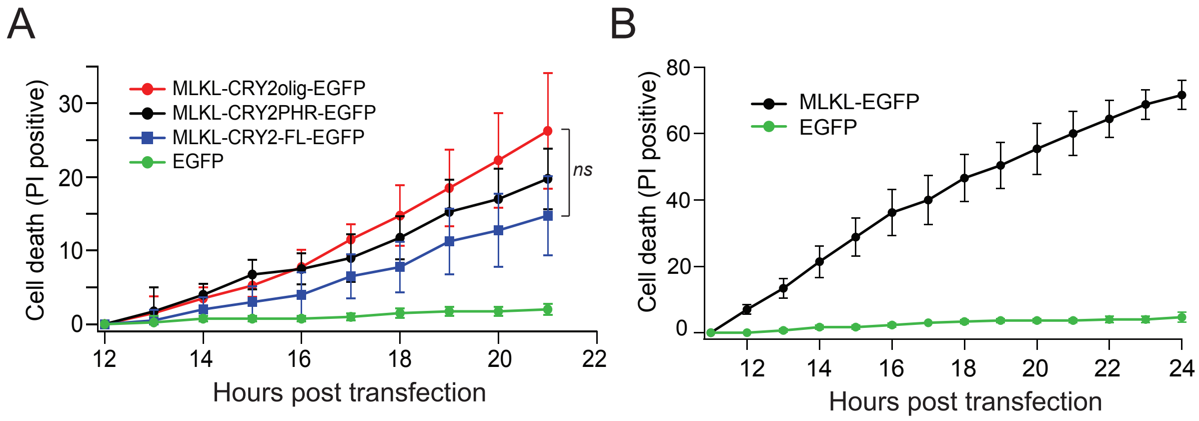

Supplementary Figure S1. Evaluation of effect of CRY2 and variants on MLKL-induced cell death. (A) Comparison of background cell death (in dark) with MLKL(1-140) fused to different CRY2 variants. HEK293T cells expressing indicated variants incubated with 1 µg/ml propidium iodide were monitored for PI staining using an Incucyte imager. Data represents average and error (s.d.), n=3. *ns, not significant, p-value < .05, two-tailed unpaired t-test.*
(B) Assessment of background cell death in cells overexpressing MLKL(1-140)-EGFP with no CRY2. Cells were treated as in (A). Substantial background cell death was observed in cells overexpressing MLKL(1-140)-EGFP, compared to an EGFP control. Data shows average and error (s.d.), n=3.

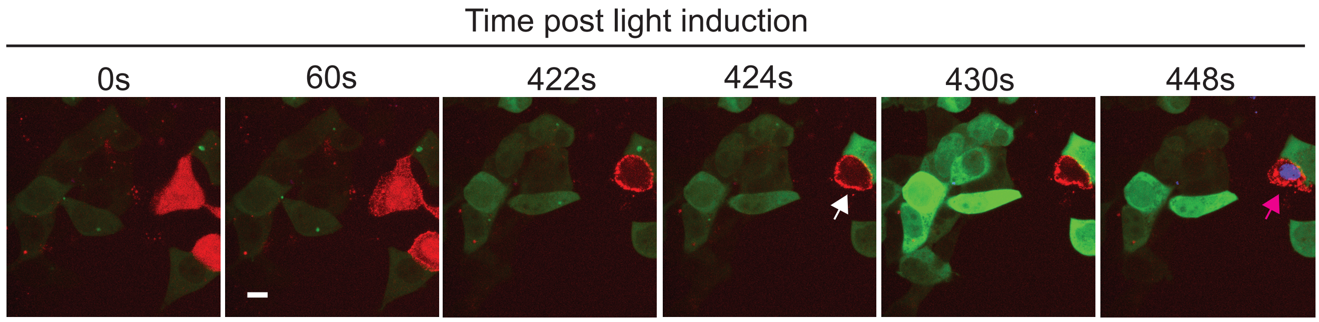

Supplementary Figure S2. Additional example of FLAG-MLKL-induced cell death signaling to bystander cells. Representative images of HEK293T cells separately transfected with a far-red labeled FLAG-MLKL-CRY2olig-miRFP670$_{nano}$ (red) or GCaMP6 (green), then mixed at a ratio of 1:5, respectively. Seconds after light stimulation, FLAG-MLKL is recruited to the PM. At 424s, a breach appears in the cell indicated (white arrow). Within seconds after rupture, the surrounding GCaMP6-expressing bystander cells show an increase in Ca2+ (GCaMP6 signal) followed by PI staining of the ruptured cell (pink arrow). *Scale bar, 10 µm.*

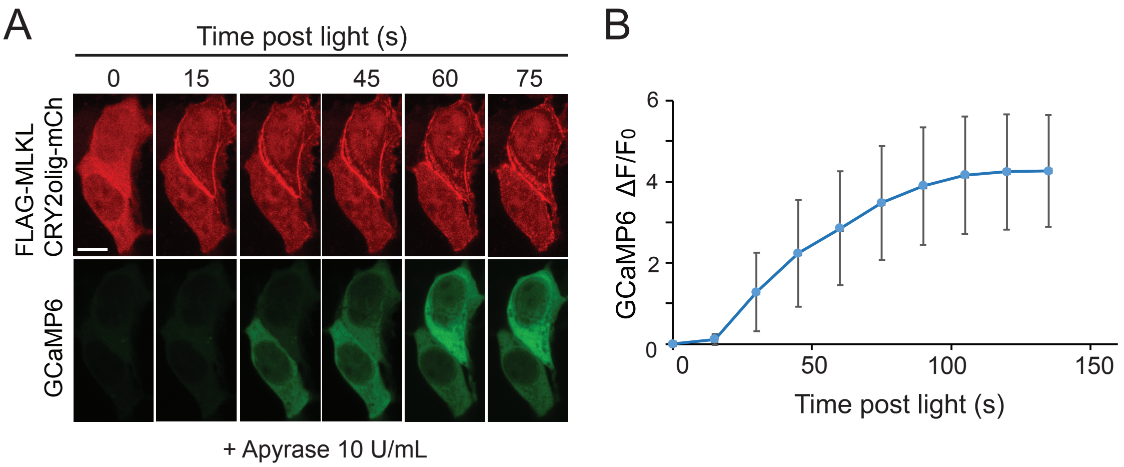

Supplementary Figure S3. Cells expressing light-activated MLKL show a rise in intracellular Ca2+ that is independent of purinergic signaling. HEK293T cells expressing FLAG-MLKL-CRY2olig-mCh and GCaMP6 were incubated with 10 U/mL apyrase then exposed to light (488 nm, 100 ms exposure every 15s). Representative images are shown in (A), with quantification of change in GCaMP6 signal in (B). Graph in (B) shows mean ± s.e.m., n=8 cells. Data are representative of 2 independent experiments. Scale bar, 10 µm.

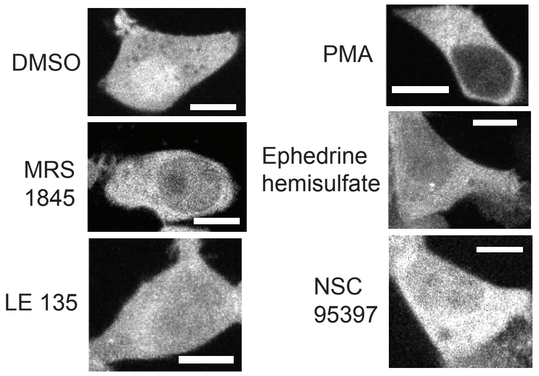

Supplementary Figure S4. Images of HEK293T cells expressing FLAG-MLKL-CRY2olig-EGFP and preincubated in dark with indicated chemicals for 1 h. FLAG-MLKL shows diffuse cytosolic localization in all examples, prior to light exposure. *Scale bars, 10 µm.*

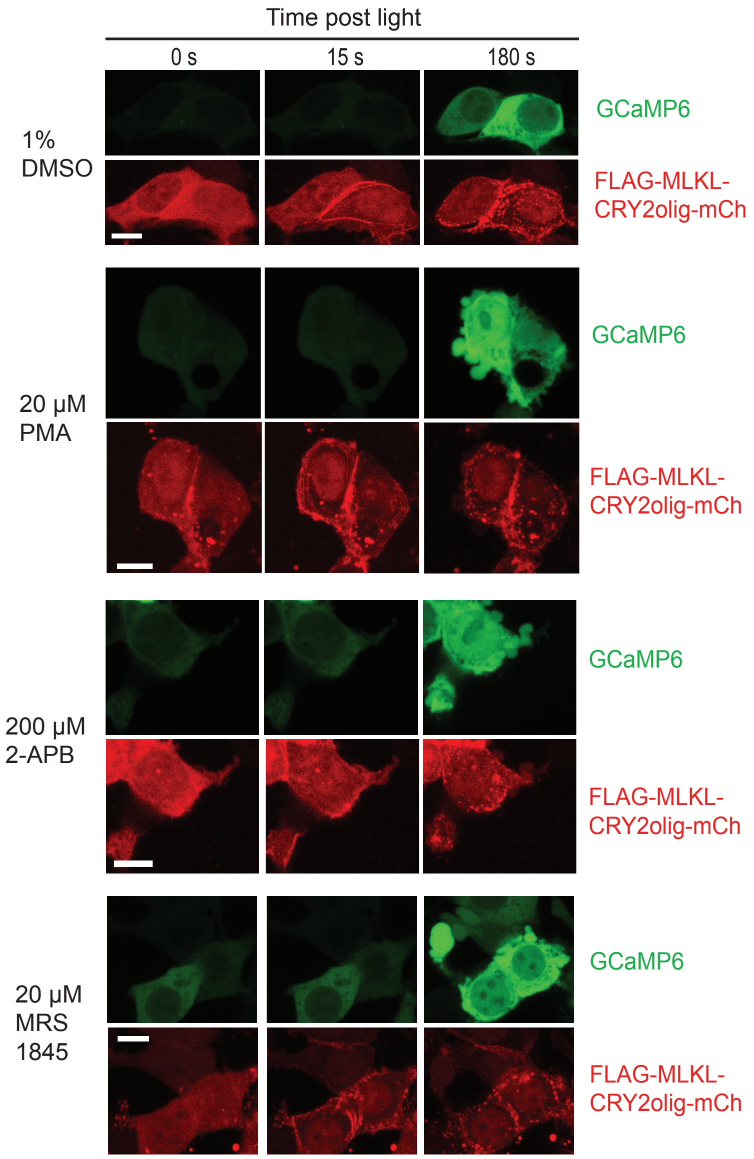

Supplementary Figure S5. Effect of compounds on light-induced MLKL cell death. Representative images of cells coexpressing FLAG-MLKL-CRY2olig-mCh and GCaMP6 (also incubated with 10U/ml apyrase) exposed to indicated compounds or 1% DMSO as a control, then stimulated with light. Cells treated with PMA, MRS1845, and 2-APB show a high degree of bleb formation 180 s post light stimulation. *Scale bars, 10µm.*

Supplementary Video legends

Supplementary Video 1. HEK293T cells expressing MLKL(1-140)-CRY2olig-mCh (and incubated with 400 nM Sytox green) exposed to light. Light exposure results in recruitment and clustering of MLKL (red) at the plasma membrane and cell death, visualized by Sytox green nuclear staining (cyan).

Supplementary Video 2. HEK203T cells expressing FLAG-MLKL and incubated with 1 μg/ml PI. Shown is a time-lapse image of HEK293T cells expressing FLAG-MLKL-CRY2olig-mCherry and incubated with propidium iodide to allow visualization of nuclear permeabilization upon cell death. Onset of light exposure at start of movie initiates FLAG-MLKL clustering and plasma membrane recruitment, resulting in PI staining of nucleus.

Supplementary Video 3. Light-triggered recruitment of FLAG-MLKL to the plasma membrane results in a rapid increase in intracellular Ca^{2+}. Shown are HEK293T cells expressing FLAG-MLKL-CRY2olig-mCh (left panel) and GCaMP6 (right panel) stimulated with blue light at the start of the movie.

Supplementary Video 4. HEK293T cells expressing FLAG-MLKL-CRY2olig-mCh (red) incubated with Cy5-Annexin-V (green) and Sytox green (magenta) probes and exposed to light at start of movie. Cells show annexin staining followed by Sytox green nuclear staining. Blue light exposure was at 00:00 (negative time frames indicate time prior to stimulation).

Supplementary Video 5. Focal stimulation of FLAG-MLKL. HEK293T cells expressing FLAG-MLKL-CRY2olig-mCh were exposed to 488nm light specifically in the area identified by the blue circle. Light exposure was at 00:00 (negative time frames indicate time prior to stimulation).

Supplementary Video 6. Light-triggered FLAG-MLKL leads to C2-PKCγ recruitment to the plasma membrane. HEK293T cells expressing FLAG-MLKL-Cry2olig-mCh (left panel) and GFP-C2-PKCγ (right panel) were exposed to 488nm light. GFP-C2-PKCγ is recruited to the plasma membrane ~100 s after light onset.

Supplementary Video 7. Signaling from dying FLAG-MLKL-expressing cell results in a spike in intracellular Ca^{2+} in surrounding cells. Shown is a composite movie of HEK293T cells separately transfected with FLAG-MLKL-CRY2olig-mCh (red) or GCaMP6 (green), then co-cultured after transfection and incubated with 1μg/ml PI. Movie begins at 8.5 minutes post initial light stimulation. A breach in the plasma membrane can be seen in the FLAG-MLKL-CRY2olig-mCh (red) cell, followed by an increase in GCaMP6 fluorescence in bystander cells, followed by nuclear PI staining of the ruptured cell.

Supplementary Video 8. Signaling from dying FLAG-MLKL-expressing cell results in transient membrane recruitment of mCh-C2-PKCγ in surrounding cells. Shown is a composite movie of HEK293T cells separately transfected with FLAG-MLKL-CRY2olig-GFP (green) or mCh-C2-PKCγ (red), then co-cultured after transfection. Movie begins at 2 min post initial light stimulation. A breach in the plasma membrane can be seen in the FLAG-MLKL-CRY2olig-EGFP (green) cell at 6 min, followed by transient membrane recruitment of mCh-C2-PKCγ in bystander cells.

Supplementary Video 9. SsrA-MLKL-CRY2olig-mCh can be recruited to the membrane repeatedly without cell death. Shown is a HEK293T cell expressing SsrA-MLKL-CRY2olig-mCh stimulated with blue light at time 0 for 30s, then left to recover in the absence of blue light (mCherry imaging only), then re-stimulated with blue light for 30s at time 17:30, then allowed to recover in the absence of blue light a second time.
